# Historical overview of development in methods to estimate burden of disease due to congenital disorders

**DOI:** 10.1007/s12687-018-0382-4

**Published:** 2018-09-12

**Authors:** Bernadette Modell, Matthew W Darlison, Joy E Lawn

**Affiliations:** 1 WHO Collaborating Centre for Community Genetics, Centre for Health Informatics and Multiprofessional Education (CHIME), University College London, London, UK; 2 London School of Hygiene & Tropical Medicine, London, UK

**Keywords:** Congenital disorders, Congenital anomalies, Genetic disorders, Rare diseases epidemiology, Mortality, Disability

## Abstract

Congenital disorders (often also called birth defects) are an important cause of mortality and disability. They encompass a wide range of disorders with differing severity that can affect any aspect of structure or function. Understanding their epidemiology is important in developing appropriate services both for their prevention and treatment. The need for epidemiological data on congenital disorders has been recognised for many decades. Here, we provide a historical overview of work that has led to the development of the Modell Global Database of Congenital Disorders (MGDb)—a tool that can be used to generate evidence-based country, regional and global estimates of the birth prevalence and outcomes of congenital disorders.

## Historical background

Congenital disorders (often also called birth defects) are an important cause of mortality and disability. They encompass a wide range of disorders with differing severity that can affect any aspect of structure or function. The need for epidemiological data on congenital disorders was recognised in the aftermath of the Second World War, when the United Nations Scientific Committee on the Effects of Atomic Radiation (UNSCEAR) was established “to collect and evaluate information on the levels and effects of exposure to ionizing radiation” (United Nations Scientific Committee on the Effects of Atomic Radiation 1977; United Nations Scientific Committee on the Effects of Atomic Radiation 1982; United Nations Scientific Committee on the Effects of Atomic Radiation 1986). Accordingly, meticulous controlled studies on the prevalence of congenital disorders in the populations of Hiroshima and Nagasaki were conducted on the assumption that an increased mutation rate would manifest as an increased birth prevalence of affected children (Schull 1958; Schull 2003; Schull et al. 1981).

At the same time, a combination of increasing technical diagnostic ability, the observed effects of rubella infection, and the thalidomide disaster of the 1960s led to increased recognition of the contribution of congenital disorders to early death and disability, the potential power of interventions and the need for ongoing surveillance. Accordingly, the World Health Organization encouraged epidemiological studies, the development of congenital anomaly registries and the construction of databases for common genetically determined disorders. The importance of surveillance was further reinforced by the thalidomide disaster in the 1960s.

### Congenital anomaly registries

Congenital anomaly registries were progressively established from the 1950s onwards. Two of the earliest registries have made unique contributions to global epidemiology. The first is the British Columbia Health Surveillance Registry, which was initiated in 1952 (Baird 1987). Whilst most congenital anomaly registries include only severe disorders associated with structural change, i.e. the “congenital malformations, deformations and chromosomal disorders” included in chapter XV11 of ICD10 (World Health Organization 1992), this registry also included single-gene disorders. The reported birth prevalences of rare single-gene disorders from this registry are relevant today, and provide the input data used in the Modell Global Database of Congenital Disorders (MGDb) (Blencowe et al. 2018; Modell et al. 2016). The second, the national Hungarian Congenital Abnormality Registry, initiated in 1962 (Czeizel 1997) was exceptional because it was partnered with a public health initiative, the Hungarian Optimal Planning Programme (Czeizel 2012; Czeizel et al. 1998). This combination enabled many invaluable studies, including a pioneering assessment of the burden of congenital anomalies in terms of years of life lost, lived with disability or lived effectively cured (Czeizel and Sankaranarayanan 1984), estimates of the power of interventions for prevention and care (Czeizel et al. 1993), and randomised controlled trials of interventions aiming to improve birth outcomes, including preconceptional supplementation with multivitamins (Czeizel and Dudas 1992) or folic acid (Czeizel et al. 1996).

Particularly important from the point of view of global epidemiology, two “umbrella registries” were initiated in 1974 to collect, standardise and harmonise birth prevalence data from individual registries and regularly publish key reference data (Moorthie et al. 2017b). The European Surveillance of Congenital Anomalies and Twins network (EUROCAT, www.eurocat-network.eu) aims to record the majority of severe congenital anomalies. The International Clearing House for Birth Defect Surveillance and Research (ICBDSR, www.icbdsr.org) collects data from countries at all levels of development, and so reports on the approximately 30% of congenital anomalies that can be reliably diagnosed around the time of birth in the absence of advanced facilities. These umbrella registries have the outstanding advantage that (when possible) they report all birth outcomes (termination of pregnancy for fetal impairment, fetal death/stillbirth, live birth), and so enable quantification of the effect of interventions that affect birth prevalence or birth outcomes.

### Databases of genetically determined disorders

Key additional resources are three databases on genetic determinants of disorders whose birth prevalence differs between populations—ABO and rhesus blood groups (Mourant et al. 1976), haemoglobin disorders and G6PD deficiency (Livingstone 1985) and prevalence of parental consanguinity (Bittles and Black 2015).

### Classical epidemiological studies

Table 1 shows classical epidemiological studies that have examined the birth prevalence of congenital disorders. These studies also have the advantage that they predate the introduction of interventions that reduce affected birth prevalence (e.g. periconceptional vitamin supplementation, termination of pregnancy for fetal impairment), or increase detection rates (e.g. routine fetal anomaly scanning, sophisticated neonatal screening). Although the range of diagnoses included differed between studies, and most studies were conducted in populations of European origin, the rates observed were broadly consistent, and were generally considered to apply worldwide (Baird et al. 1988).

**Table 1:** Classical studies of the birth prevalence of congenital disorders

Source	Chromosomal disorders	Congenital malformations	Rare single-gene disorders
Neel (1958)		+	
Stevenson (1959)	+	+	+
Stevenson et al. (1966)		+	
Trimble and Doughty (1974)	+	+	+
Myrianthopoulos and Chung (1974)		+	
Ash et al. (1977)	+	+	+
Hook and Hamerton (1977)	+		
Carter (1977)			+
Czeizel and Sankaranarayanan (1984)	+	+	

All studies include only disorders that cause death or disability in the absence of intervention

AC Stevenson was the first to attempt to measure “the load of hereditary defects in human populations”, based on the number and age distribution of patients referred to his Northern Ireland clinic (Stevenson 1959). This study includes valuable estimates of birth prevalence and early mortality at a time when only supportive care was available. It led the WHO to conduct a comparative study of the birth prevalence of selected congenital anomalies in 24 centres representing all WHO regions, to review the global prevalence and management of haemoglobin disorders and to publish three technical reports (Stevenson et al. 1966; World Health Organization 1966; World Health Organization 1972).

## Towards development of a global epidemiological picture

In the early 1980s, Anver Kuliev, then director of the WHO Hereditary Diseases Programme, reviewed the available data and identified the three key elements of Community Genetics—epidemiology, audit of the effect of interventions (surveillance) and information and education (World Health Organization 1985). Accordingly, he initiated development of a global epidemiological picture, starting with country-specific estimates for the haemoglobin disorders (Modell and Darlison 2008). The work was continued with support from the Wellcome Trust and the WHO Office for the Eastern Mediterranean Region (Alwan et al. 1997; Christianson and Modell 2004; Modell 1992). The resulting estimates contributed to several WHO reports (World Health Organization 2000; World Health Organization and March of Dimes 2006), provided the quantitative basis for the influential March of Dimes Global Report on Birth Defects (Christianson et al. 2006) and contributed baseline estimates for the “Born Healthy”(http://www.bornhealthy.org) Needs Assessment Toolkit (Nacul et al. 2014). WHO endorsement of the March of Dimes estimates led in turn to inclusion of congenital disorders in the Global Burden of Disease study (Lopez et al. 2006), and a Resolution on Birth Defects by the World Health Assembly (World Health Organization and 2010).

The Executive Board’s first recommendation to the World Health Assembly was “to promote the collection of data on the global burden of mortality and morbidity due to birth defects”, and the resolution included the recommendation to “resolve currently divergent opinions on the health burden of both environmental and constitutional birth defects”. We therefore formed an informal international expert group to develop and review methods for estimating birth prevalence and outcomes of congenital disorders, compliant with the recommendations of the Guidelines for Accurate and Transparent Health Estimates Reporting (GATHER) Working Group (Stevens et al. 2016).

### The Modell Global Database of Congenital Disorders

**Fig. 1:**
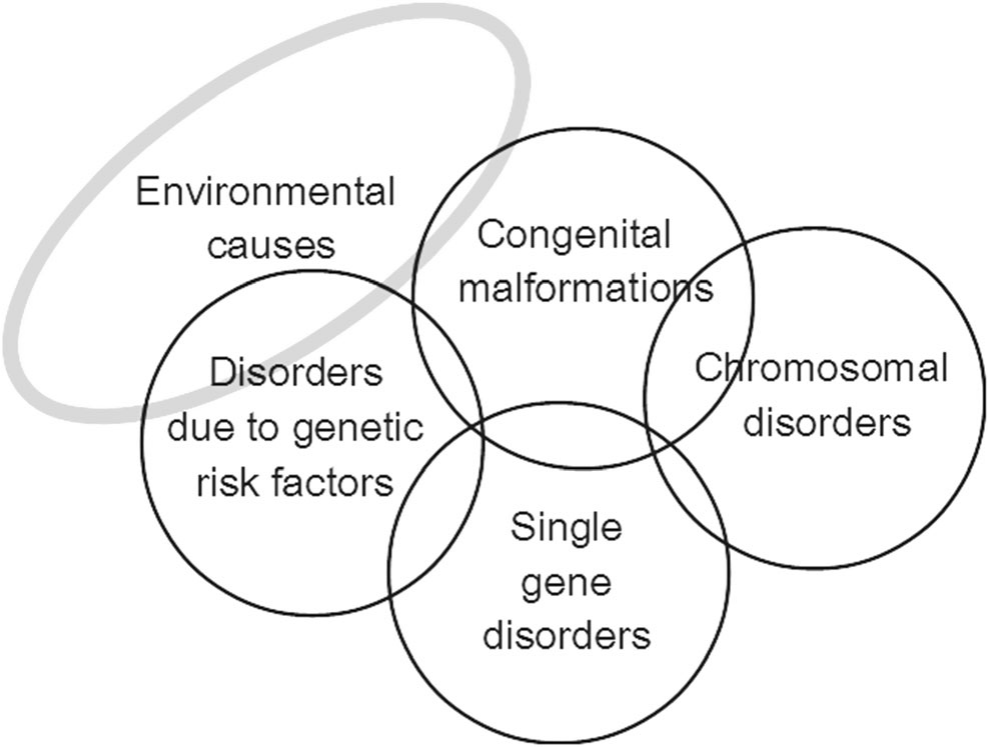
The main groups of congenital disorders. The image is notional: there is no relation between frequency and the size of the circles, or actual extent of overlap between categories. MGDb currently includes only the groups whose birth prevalence is relatively constant or can be calculated, namely chromosomal disorders, early-onset congenital malformations, single-gene disorders and two examples of common early-onset disorders due to genetic risk factors—rhesus haemolytic disease and neonatal jaundice due to glucose-6-phosphate dehydrogenase deficiency.

Congenital disorders can be divided into five groups: environmental disorders (due to maternal exposure to, e.g. infection or other hazards), chromosomal disorders, congenital malformations, single-gene disorders and disorders due to common genetic risk factors (Fig. 1). In the absence of intervention, the birth prevalence of four of these groups is largely determined by endogenous causes, so is relatively constant for any given population, and may be called their *baseline birth prevalence.* Sufficient data is available to obtain country-specific estimates of baseline birth prevalence for these four disorder groups (Modell et al. 2016; Moorthie et al. 2017a).

The availability of a baseline birth prevalence is an unusual, possibly unique, characteristic. It offers exceptional advantages from an epidemiological point of view because once it is known, it can be related to available demographic and survival data and the observed effects of interventions, to reach country-specific estimates of the distribution of actual outcomes, including termination of pregnancy, fetal death, under-five death, disability and cure. Furthermore, the sum of outcomes must fill the original “envelope” of baseline birth prevalence. That is, these disorder groups can be handled as closed systems. In view of this important characteristic, these four groups are here collectively called *constitutional congenital disorders*.

The MGDb exploits this exceptional characteristic to generate evidence-based country, regional and global estimates of the birth prevalence and outcomes of constitutional congenital disorders that can be used as a starting point for service planning.

Currently, MGDb does not include congenital disorders caused by environmental risk factors such as maternal infection, malnutrition or exposure to teratogens. This is not to detract from the relevance of “environmental” congenital disorders. They have high priority because they can be largely prevented by basic public health interventions including sanitation, immunisation, nutritional supplementation, restriction of exposure to teratogens and diagnosis and treatment for the mother before or during pregnancy, and so occupy an important position in WHO recommendations for pregnancy care (World Health Organisation 2013). They are not currently included in MGDb because their baseline birth prevalence is determined by exposure to risk and so varies with place, time and deployment of interventions. Ongoing surveillance or periodic surveys are required to follow their epidemiology but resources for surveillance are most limited in the countries where they are most prevalent. Therefore, insufficient country- and time-specific observational data are available to permit the form of modelling used in MGDb. Should sufficient data become available, they might be included in the future.

In building MGDb, we developed a subset of epidemiological methods specific to congenital disorders that are simple enough for use by non-specialist health professionals. The articles in this special issue of the Journal of Community Genetics provide an introduction to these methods. Of equal importance, we encountered many examples of ill-defined or ambiguous terminology, so that it was necessary to establish precise definitions of all terms used. Selected country-specific outputs, a full description of the methods and the terminology used are available online at http://discovery.ucl.ac.uk/1532179/ (Modell et al. 2016).

## Other sources of data on epidemiology of congenital disorders

The Global Burden of Disease (GBD) study has also published country-specific estimates of mortality and disability due to congenital anomalies, haemoglobin disorders and G6PD deficiency. The methods used by GBD differ from those used in MGDb and this leads to wide differences between the estimates. For example, the omission by GBD of stillbirths and terminations of pregnancy for fetal impairment leads to serious underestimation of the burden of congenital anomalies in high-income settings (Boyle et al. 2017), whilst the lack of accurate cause of death data in low- and middle-income countries leads to serious underestimation of early mortality due to congenital anomalies by the GBD (Boyle et al. 2017; Moorthie et al. 2017a). In addition, the fact that estimates for early-onset single-gene disorders, rhesus haemolytic disease and kernicterus due to G6PD deficiency are included in MGDb but not in the GBD widens the ostensible difference between the estimates.

### Conclusions

Congenital disorders continue to be important contributors to mortality and disability. The need for epidemiological data in this field has been recognised for decades. Policy and service development are reliant on such data to assess the burden of disease and the impact of interventions. Efforts such as the establishment of registries and research studies help to generate evidence to support policy, but there is still a lack data from low- and middle-income countries. Simple methods that enable policymakers to make up for this deficiency are needed as a starting point.
